# A high transmission tender X-ray monochromator employing a matched pair of multilayer grating and mirror

**DOI:** 10.1107/S1600577525009968

**Published:** 2026-01-01

**Authors:** David Dennetiere, Thierry Moreno, Blandine Capitanio, Muriel Thomasset, Franck Delmotte, Regina Soufli, Catherine Burcklen, Evgueni Meltchakov, Gianluca Ciatto, Philippe Fontaine, Pascal Mercère, François Polack

**Affiliations:** a Synchrotron Soleil L’Orme des Merisiers, Départementale 128 91190Saint-Aubin France; b Centre de Nanosciences et de Nanotechnologies CNRS, Université Paris-Saclay 91127Palaiseau France; c Institut d’Optique Graduate School, CNRS, Laboratoire Charles Fabry CNRS, Université Paris-Saclay 91127Palaiseau France; d Lawrence Livermore National Laboratory 7000 East Avenue Livermore CA94550 USA; RIKEN SPring-8 Center, Japan

**Keywords:** multilayer grating monochromator, tender X-rays, diffraction efficiency modeling, design and tuning optimization

## Abstract

It is now well established that multilayer coated gratings may offer high diffraction efficiencies over the tender X-ray range, from about 1 keV to 5 keV, covering the gap between single layer coated grating monochromators and crystal monochromators. Nevertheless, few beamlines in the world are using such gratings in their monochromator. The successful implementation of a multilayer grating monochromator requires producing a matched pair of a multilayer grating and a multilayer mirror, and this matching is not straightforward as it must account for different incidence angles and refraction effects on each element. Here we review the realization of the multilayer grating monochromator of the SIRIUS beamline which has been successfully in service for several years. We show how, by alternating computer simulation with our diffraction code and measurements, we could optimize the monochromator transmission on a very wide energy range. After the grating was coated, it was found that the angle of optimal efficiency versus photon energy was significantly different from what was predicted by a simple conformal model of binary layers. Layer interdiffusion and profile smoothing during the deposition process must be added to the multilayer model to reproduce the measured data. The critical adjustment of the mirror multilayer period is achieved by the lateral translation of the mirror, which was given a small transverse period gradient. The monochromator is thus providing high transmission efficiency in the 1 to 5 keV energy range, more than 30% over 2.5 keV and up to 46% at 4.6 keV.

## Abstract 2

Monochromators based on multilayer gratings can provide a very high throughput on an extended energy range (1 to 5 keV) in the tender X-ray domain. A multilayer mirror must be associated with the grating to achieve a fixed exit direction. We show how important an accurate modeling of the reflectivity of these two elements is to achieve both an optimal design and an optimal tuning.

## 1. Introduction

Most X-ray spectroscopy techniques used at synchrotron radiation facilities require the radiation to be monochromated over a large energy range. This is done by reflective gratings in the soft X-ray range and crystal monochromators in hard X-rays. With reflective gratings coated with a single metallic layer, there is no overlap between the two ranges, since the reflectivity of metal coatings steeply decreases for energies over 1500 eV, while Si crystals, which can sustain the flux of such sources, have limitations below 3 keV, instabilities at near normal incidences, and loss of p-polarization for incidences around 45°. The tender X-ray domain of 1–4 keV, which provides many spectroscopic opportunities on *K*-edges of Al, Si, P, S and K or *L*-edges on Nb, Mo, In and Sb, cannot be accessed continuously.

However, it was shown nearly 20 years ago, both by simulations using rigorous coupled wave approximation [RCWA (Montiel & Neviere, 1994 ▸; Popov & Nevière, 2001 ▸)] and by experiments, that enhanced diffraction efficiencies can be achieved in tender X-rays with multilayer (ML) coated gratings (MLG) (Polack *et al.*, 2007 ▸). This enhancement happens when the periodic modulation created along the surface by the grating substrate and the periodic modulation perpendicular to the surface imposed by the ML combine to allow the propagation of only one diffracted wave. For a laminar grating the optimal diffraction conditions are found when the depth of the rectangular groove profile is exactly half of the ML period, alternating periodically the distribution of the ML materials, an arrangement which was called alternate multilayer grating (AMG) (Lagarde *et al.*, 2013 ▸; Choueikani *et al.*, 2014 ▸). The pseudo-crystal selectivity given by the 2D structure also explains the observed efficiency of etched multilayer gratings around 1500 eV as acknowledged by Kozhevnikov *et al.* (2010 ▸), despite the name lamellar amplitude multilayer grating (LMAG) given earlier by J. M. André and co-workers to this structure (André *et al.*, 2001 ▸; Benbalagh *et al.*, 2005 ▸).

The deposition of an ML coating on a sawtooth profile yields a so-called blazed multilayer grating (BMG). BMGs can offer a slightly higher diffraction efficiency than AMGs, due to their asymmetric structure. Production of conformal BMGs with minimal rounding effect of material deposition has been extensively studied by D. Voronov *et al.* (Voronov *et al.*, 2012 ▸; Voronov *et al.*, 2013 ▸; Voronov *et al.*, 2014 ▸; Voronov *et al.*, 2016 ▸). More recently, I. V. Kozhevnikov, Q. Huang and co-workers have extensively studied the ML coated gratings from LMAG (Yang *et al.*, 2015 ▸) to AMG (Yang *et al.*, 2017*a* ▸; Wen *et al.*, 2024 ▸) and BMG (Sokolov *et al.*, 2019 ▸; Huang *et al.*, 2020 ▸; Wen *et al.*, 2025 ▸), developing an analytical theory for ‘single-order’ gratings.

All these studies are of course motivated by their potential use in spectrometers and monochromators (*e.g.* Yang *et al.*, 2017*b* ▸). However, despite the number of MLG related publications, very few instrument realizations have been reported in the literature. Besides the three monochromators in operation on SOLEIL synchrotron beamlines for many years, LUCIA with an LMAG (Vantelon *et al.*, 2016 ▸), DEIMOS (Ohresser *et al.*, 2014 ▸; Choueikani *et al.*, 2014 ▸) and SIRIUS (Ciatto *et al.*, 2016 ▸; Hemmerle *et al.*, 2024 ▸) with AMGs, we only know of the U41 undulator beamline of BESSY II that uses a BMG (Werner *et al.*, 2023 ▸). A grating monochromator for a synchrotron beamline requires two reflections, one on the grating and another, on a mirror, to bring the beam back to a fixed, and almost horizontal, exit direction. In tender X-rays, the large deviation produced by the MLG can only be compensated by an ML coated mirror having almost the same ML period. The performance of an MLG monochromator hence depends on the proper match between an ML grating and an ML mirror.

Since the incidence angles onto the grating and the mirror are different, so are the resulting refraction angles inside the two thick coatings. As a consequence, matching the mirror ML to the grating’s one is not as straightforward as using the same multilayer with the same period on both. Optimal match requires extensive measurements and simulations in order to achieve the best performance on the extended energy range into which an ML monochromator can work. In this article we review the procedure that was used to optimize the efficiency of the monochromator of the SIRIUS beamline. We will follow the stages of development starting from the design principle of the grating in Section 2, then, prior to finalizing the ML mirror parameters, the metrology of the coated grating alone in Section 3. The results of these measurements pushed forward towards significantly improving the model of the ML structure, departing from the basic conformal replication of the grating profile by homogeneous layers with perfect interfaces, in Section 4. The final parameters of the ML mirror and of the monochromator control were then derived from this refined model, Section 5, leading to the instrument which has been in operation since 2017.

## 2. Design principles

The grating of the SIRIUS beamline was designed to operate at energies from 1.1 to 4.5 keV in its first external diffraction order (grazing incident angle greater than the diffracted beam grazing angle). It is used in alternance with an Si111 crystal monochromator which provides higher energies up to 13 keV. The two monochromators are the first elements of the beamline and share the same refocusing optics (Ciatto *et al.*, 2016 ▸). They are directly illuminated by the undulator source located 20 m upstream.

The grating substrate is a custom-made lamellar ion-etched grating from HORIBA France SAS, with a period of 416.7 nm, and a shallow groove depth. The grating profile was measured before and after coating with an atomic force microscope (AFM) in the SOLEIL metrology cleanroom. The parameters of the uncoated grating profile are given in Table 1 ▸.

The grating period is varied along the length of the grating so as to compensate for the aberrations of asymmetric reflection of a spherical wavefront (Reininger & de Castro, 2005 ▸; Reininger, 2011 ▸). The divergence of the incoming beam is small and so is the required varied line spacing (VLS) correction. The design of the ML coating does not take this feature into account as the overall period change on the 60 mm useful length of the grating is very small. The measurements shown later in this article are made using a few millimetres X-ray footprint so that this variation is negligible.

The Cr/B_4_C material pair was chosen for the ML coating, since both Cr and B_4_C are free of strongly absorbing edges in the 1000–4500 eV domain, while preserving a good index contrast over the whole domain and having a proven lifetime stability (Burcklen *et al.*, 2016 ▸; Burcklen *et al.*, 2019 ▸). The optimum ML period was at first chosen to be twice the grating groove depth with a thickness ratio Γ [Γ = *d*_Cr_/(*d*_Cr_ + *d*_B_4_C_)] of 0.5 so that the resulting structure would have a checkerboard-like section, thus defining Bragg planes at angles θ(*m*,*n*) with respect to the grating surface,
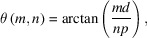
*m* and *n* being, respectively, the orders of diffraction of the ML and the grating, with *d* the multilayer period and *p* the grating groove period.

Reflectivity simulations were carried out with our electromagnetic propagation code *CARPEM* (Mirone *et al.*, 1998 ▸). The code is based on RCWA for short distance propagation, coupled with a reflectivity matrix propagation algorithm for larger thicknesses. It considers purely dielectric media (no currents) which is a valid approximation in the X-ray domain. In the original version used for designing the grating parameters, the modulated region is decomposed into layers, each of which contains two alternate rectangular blocks of materials. All boundaries are sharp.

The simulation results showed that decreasing the Γ ratio of the ML leads to a higher peak reflectivity and a narrower bandwidth. We chose to maximize the reflectivity even if this meant a condition on the incident angle (α) and the output angle (β) to be determined later on. In order to minimize the risks and instabilities inherent to long deposition times a compromise was searched between the number of layer pairs, the Γ ratio and the reflectivity. As B_4_C has a slow deposition rate compared with Cr, a low Γ ratio increases the deposition time.

**Figure 1:**
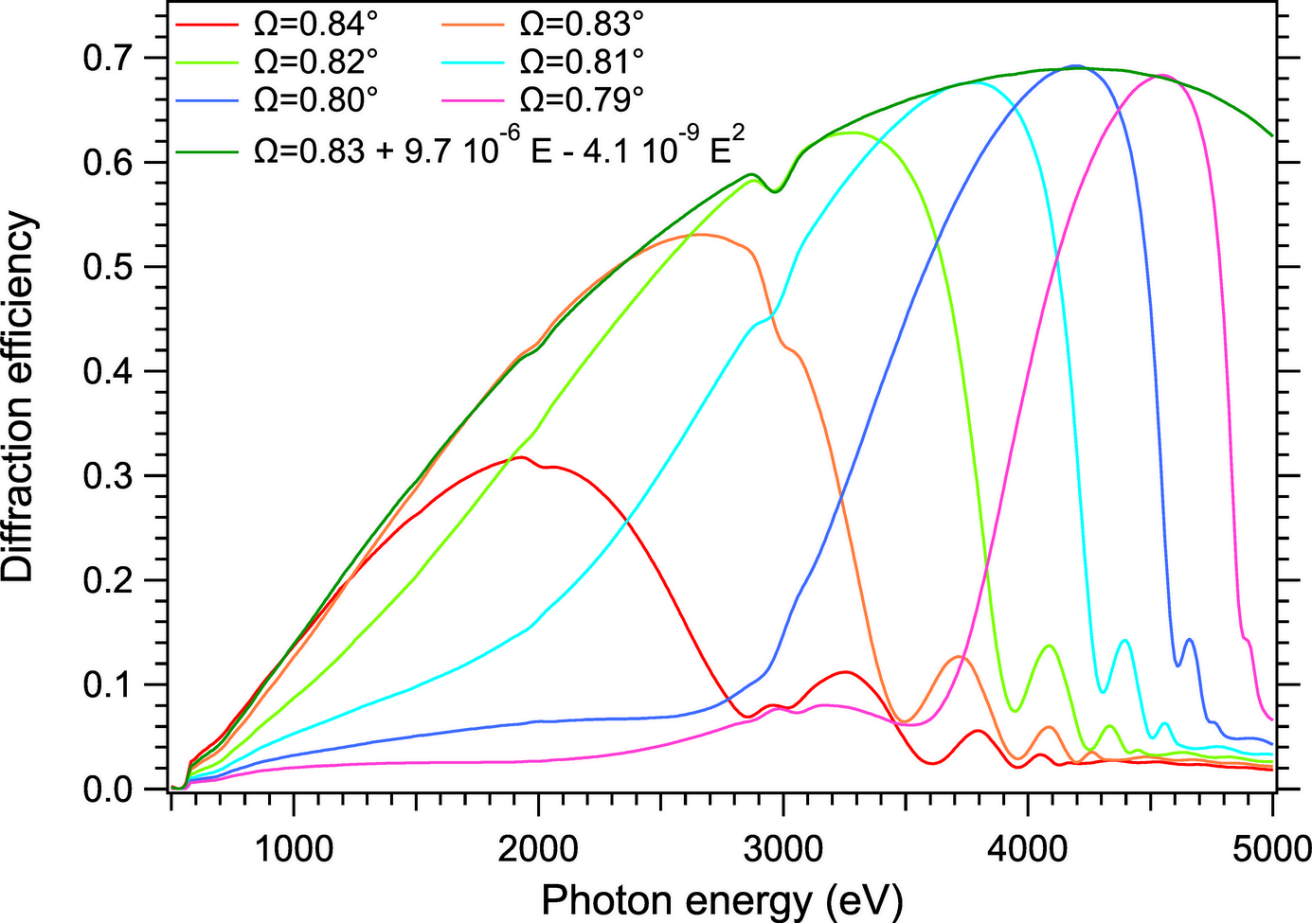
First order of the grating for different Ω values, estimated with a model of two layers per ML period and abrupt interfaces.

A first design was then simulated with a groove depth of 3.3 nm, a 2400 mm^−1^ groove density, and 35 Cr/B_4_C periods of 6.6 nm each and a Γ ratio of 0.38, allowing for almost saturated reflection at 4 keV, while keeping a reasonable deposition time. It has been shown (Choueikani *et al.*, 2014 ▸) that driving the grating angles using a constant value for 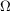 = 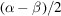 is a good approximation of the Bragg condition. However, the average index inside the grating structure is not exactly 1 so that a slight energy dependent correction must be applied to keep the reflectivity at its maximum value. We found that the Ω = *f*(*E*) law of maximum efficiency, where *E* is the photon energy, is well approximated by a polynomial function, as shown in Fig. 1 ▸.

Two glitches are noticeable around 2 and 3 keV, as the Ewald diffraction sphere passing by node (0, 0) and node (1, 1) of the grating reciprocal space also intersects the truncation rod of another node, and thus three waves instead of two can propagate in the modulated volume of the 2D grating (Van Der Laan & Thole, 1988 ▸).

## 3. Efficiency measurement

**Figure 2:**
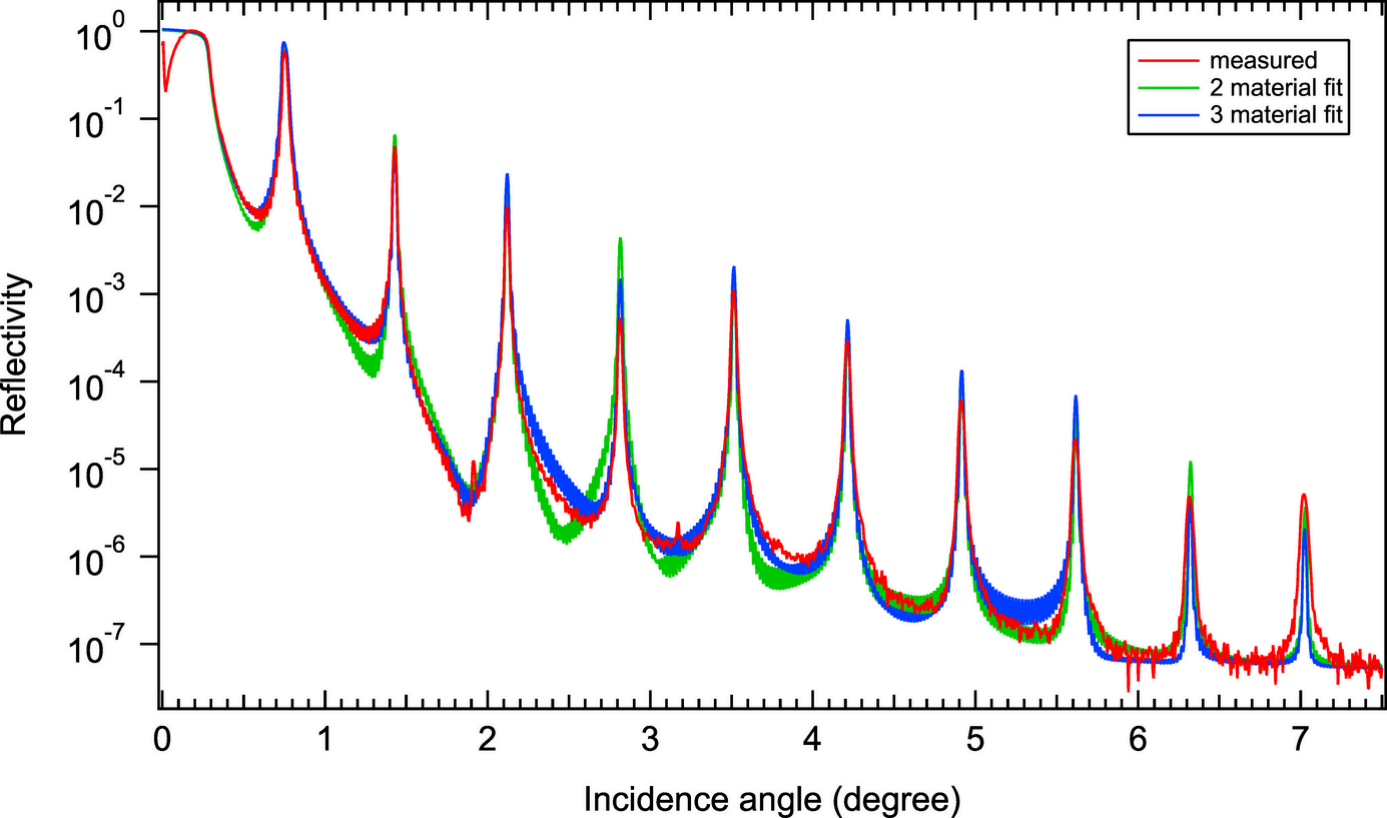
Grazing incidence reflectometry with Cu *K*α X-rays of the ML coated grating in the direction parallel to the grooves. The fit is done with *LEPTOS* software, considering two or three layers per ML period and asymmetric layer interfaces across the ML coating (see Table 2 ▸).

The Cr/B_4_C ML coating was deposited by magnetron sputtering at the Laboratoire Charles Fabry (LCF) using a Plassys MP800 deposition system. A full description of the deposition system has been given by Gautier *et al.* (2005 ▸) and deposition parameters used for similar Cr/B_4_C MLs by Burcklen *et al.* (2016 ▸). Trial coatings with the ML parameters of the design mentioned in the previous section were first made on unstructured plane substrates and measured using Cu *K*α grazing incidence reflectometry using a Bruker Discover D8 diffractometer. The diffractometer experimental setup can be found in the paper by Choueikani *et al.* (2013 ▸). On finished gratings, measurements in a plane parallel to the grating lines appear to yield reliable results (see Appendix *A*[App appa]). The measurements (Fig. 2 ▸) were then fitted with the multilayer coating retrofitting algorithm *LEPTOS* (Ulyanenkov, 2004 ▸). The density of the layers was given a slightly different value from the bulk density, as experience has shown that the deposited layers are lighter than the bulk. A surface layer of B_2_O_3_ was also added, to take surface oxidation into account (Table 2 ▸) (Burcklen *et al.*, 2016 ▸; Burcklen *et al.*, 2019 ▸).

The models inferred by *LEPTOS* showed a rather high interfacial roughness of the order of 0.68 nm, *i.e.* 30% of the Cr layer. It is important to note also that the interfacial roughness is asymmetrical, *i.e.* very high when B_4_C is deposited over Cr and rather low otherwise (0.21 nm). Note that, in the model, the roughness parameter actually describes a smooth transition of the average index between two layers. As material interdiffusion produces a similar index transition, the two phenomena are not distinguished, and a unique parameter can account for both.

Initially our propagation code *CARPEM* did not take interfacial diffusion/roughness into account but assumed a stack of layers of fixed composition and perfect interfaces. We had to try to find other models fitting the measured reflectance. The experience of the LCF team led us to consider two other models fitting the reflectance very well and presenting very low roughness.

The first model retrofitted with the *LEPTOS* software considers a third layer (interlayer, IL) at the B_4_C-on-Cr interface, the index of which is that of a layer stoichiometrically described as Cr_11_B_8_C_2_. The second one, inferred from simulation with David Windt’s code *IMD* (Windt, 1998 ▸), represents this third layer as a mean index between Cr and B_4_C. In both codes (*LEPTOS* and *IMD*), the Nevot-Croce model is used to describe interfacial imperfections (roughness and/or diffusion). Since these two models are inferred from the same reflectivity measurement, when reinjected in *CARPEM* they all yield the same maximal efficiencies, but the Ω angles for which these efficiencies are reached are different. The ML model parameters are given in Table 2 ▸.

**Figure 3:**
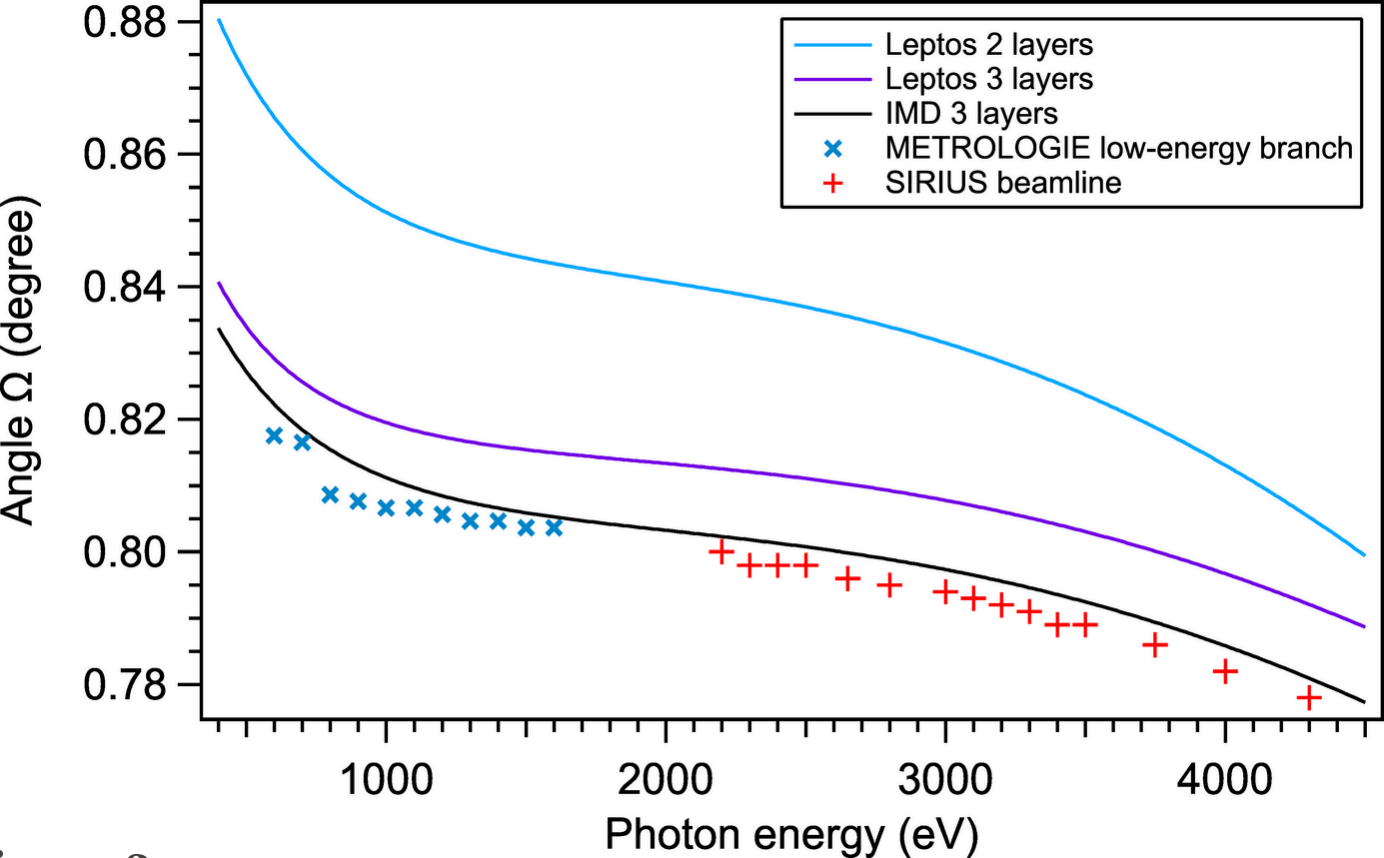
Comparison between the simulated Ω for which the maximal efficiency is reached and the actually measured Ω versus photon energy.

The coated grating was measured at the SOLEIL synchrotron on the SIRIUS beamline using the Si111 double crystal monochromator (DCM) for the 2200–4300 eV energy range, then on the METROLOGIE beamline’s soft X-ray branch (Idir *et al.*, 2010 ▸) for the 600–1500 eV energy range. The measurement was made with a detector aperture allowing for the whole order of diffraction to be integrated while minimizing the noise. The optimal Ω angles were measured while varying both α and β according to the grating law and taking the value for which the efficiency was the highest. The resulting values of Ω are shown in Fig. 3 ▸. This measurement shows that the behavior of the AMG grating is very close to the IMD three-layer model.

This means that what was, at the beginning, retrofitted in two layers as a high roughness is most likely the result of the diffusion of the Cr atoms across the surface separating the materials. Such a phenomenon is often observed on coated surfaces for which an adhesive Cr layer has been used (George *et al.*, 1990 ▸), and asymmetrical interfaces have already been reported for other Cr-based multilayers (Choueikani *et al.*, 2013 ▸). Moreover, the asymmetry of the Cr/B_4_C interfaces is consistent with further observations on the same pair of materials (Burcklen *et al.*, 2016 ▸; Burcklen *et al.*, 2019 ▸). Our assumption is that the deposition of B_4_C over a Cr layer yields a significant interdiffusion of B_4_C particles into the Cr layer, since Cr atoms are highly mobile. On the contrary, the B_4_C surface forms a relatively solid barrier into which Cr atoms will not penetrate when Cr is sputtered onto B_4_C.

**Figure 4:**
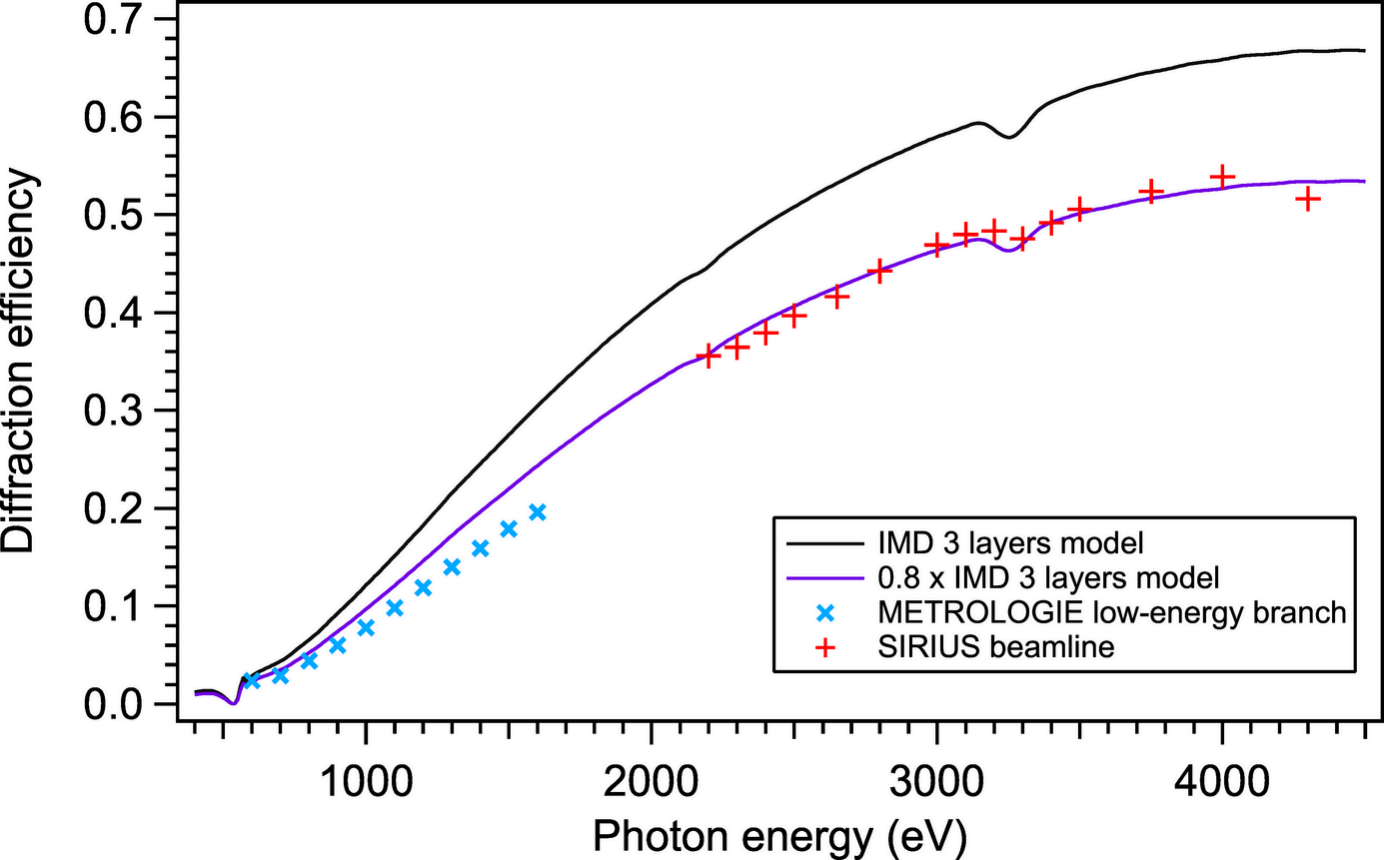
Comparison between the simulated efficiency of the first order of the grating and the efficiency measured on the METROLOGIE (soft X-ray branch) and SIRIUS beamlines.

Though the three-layer model reflects well the angular position of the measured peak efficiency, the peak efficiencies themselves are significantly different. Fig. 4 ▸ compares the efficiency curve computed by *CARPEM* from the IMD three-layer model, with actual measured values. The general trend of reflectivity versus energy is respected, but a correction factor of 0.8 must be applied to *CARPEM* simulated reflectivity to match the measurements. The same factor of 0.8 was observed earlier in *CARPEM* simulations (Choueikani *et al.*, 2014 ▸).

## 4. A refined grating model

The observed discrepancy between simulated and measured diffraction efficiencies showed the need for a more accurate description of the grating structure in *CARPEM* input data. This model must take into account the real grating profile, determined with AFM, onto which the multilayer is deposited, and the multilayer composition versus deposited thickness. This vertical material distribution can be determined by grazing incidence X-ray reflectometry in the direction parallel to the grating grooves (see Section 3).

Because the height modulation of the substrate grating is small with respect to the grating period, the profile slopes remain low and we may expect a conformal replication, that is an identical vertical distribution of materials over any point of the grating. It would be therefore easy to compute the Fourier expansion of index distribution in any plane parallel to the average grating surface as required by the RCWA algorithm of *CARPEM*.

**Figure 5:**
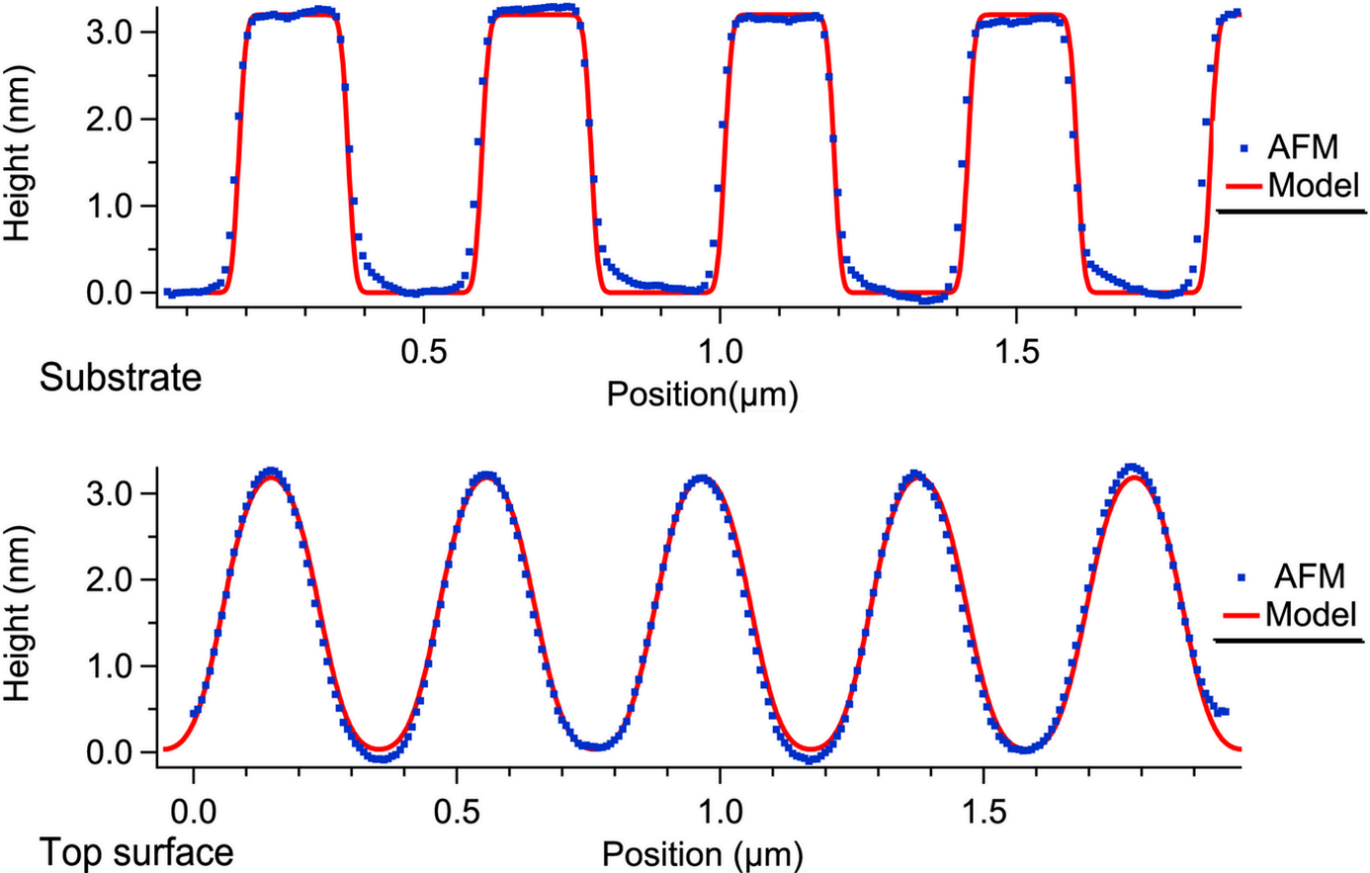
AFM measurements in the central area of the grating before and after multilayer coating. The two profiles were recorded near the grating center and they are averaged over a line length of ∼0.2 µm. The measured profiles (blue dots) are compared with modeled profiles (red lines) computed with the parameters of Table 3 ▸.

The AFM measurements of the grating surface before and after coating, Fig. 5 ▸, show that the conformal assumption is not fully valid. The coating is thicker in the regions of concave curvature and thinner in the regions of convex curvature. The phenomenon is akin to a smoothing effect which is well documented for B_4_C layers where it is related to the internal stress of the ML (Pradhan *et al.*, 2018 ▸; Wu *et al.*, 2019 ▸). The origin of a local material redistribution during layer growth must be sought in the physics of the deposition process, and the material properties should play a role since, in other situations, some materials are observed to grow thicker on convex rather than concave area.

From these considerations we propose a simple model of the multilayer growth where the average growth rate is linearly dependent on the local curvature,
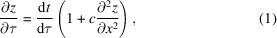
where *z* is the ordinate of the growth interface, *t* is the time dependent deposited thickness on a flat surface, *x* is the position along the grating, τ is the time, and *c* is the linear constant of growth versus curvature dependence.

The profile of the grating is given by its Fourier expansion,
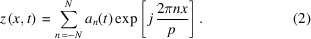
Derivation of (2) using (1) then yields
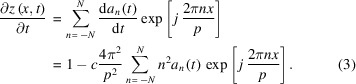
Integrating, we get from identification,
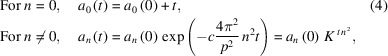
with
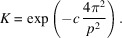
The constant *K* is determined to fit the measured profiles before and after deposition.

The substrate profile over one period is modeled by a rounded box function, the Fourier expansion of which is given by
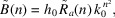
where 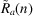 is the Fourier expansion of the unit rectangular boxcar function of duty cycle *a*, and the other parameters are defined in Table 3 ▸.

Fig. 5 ▸ shows, on top of the measured AFM profiles, the same profiles simulated with the values of Table 3 ▸.

**Figure 6:**
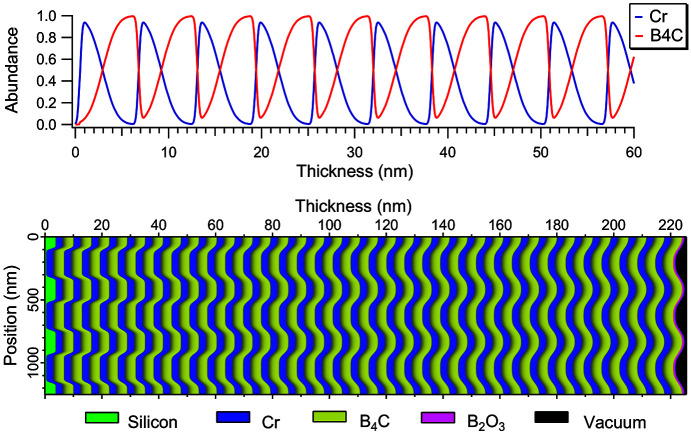
Distribution of materials inside the modulated volume. Top panel: relative abundance of Cr and B_4_C *versus* deposited thickness (over flat surface areas). Bottom panel: material distribution map of 2.5 grating periods (left axis) inside the multilayer showing the damping of the shape as a function of thickness (top axis). The substrate, in green, is on the left and vacuum, in black, on the right.

To model the vertical index distribution in the multilayer stack we took into consideration the results of Section 1. The transition between materials is figured by a smooth change of their relative abundances in the form of two complementary error functions, representing both interdiffusion and roughness. Since the better match of Ω angles was found with an interlayer, the index of which is the average of Cr and B_4_C, we also represent the interface B_4_C over Cr as a simple error function transition between these two materials. The σ value of this transition was taken as 1.2 nm. This value is supported by other studies (Burcklen *et al.*, 2016 ▸; Burcklen *et al.*, 2019 ▸) which have reported transition layers of thickness varying from 1 to 1.5 nm when B_4_C is deposited on Cr layers of increasing thickness. The parameters of the model are given in Table 4 ▸; material densities are those of Table 2 ▸, and the resulting material distribution is shown in Fig. 6 ▸. The simulation by *CARPEM* of the Cu *K*α reflectivity of this ML structure is in good agreement with the simulations of Section 1.

We refer to the combined model of continuous material distribution between layers and curvature dependent growth as GRD (Graded material Distribution), to emphasize the index continuity in the modeled grating structure.

The RCWA algorithm of *CARPEM* requires knowledge of the Fourier transform of the optical constants (ɛ or 1/ɛ) in any plane parallel to the surface. The parameters of the GRD model are pre-processed to provide maps of the abundance of each of the material present in the structure. These maps, which are sampled on a regular grid with a typical step of 0.1 nm in thickness, are input to *CARPEM* to allow a fast interpolation of the index at any depth.

**Figure 7:**
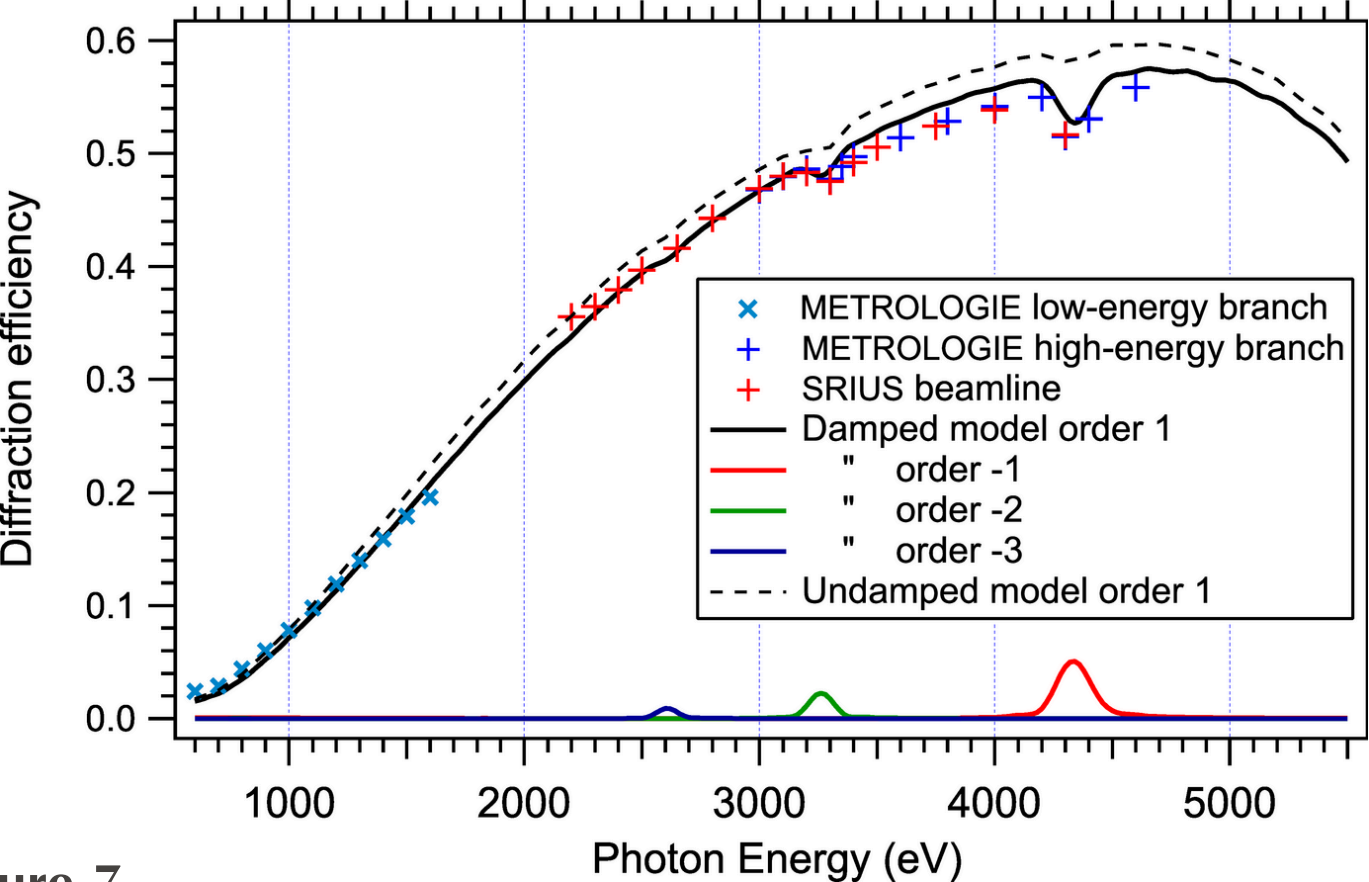
Comparison between the GRD undamped model, the GRD damped model and measurements made on the SIRIUS and METROLOGIE beamlines at SOLEIL. The glitches are well predicted by the damped GRD model and correspond to energy leaks into other orders when the Bragg condition is simultaneously satisfied for two vectors of the reciprocal space.

Fig. 7 ▸ shows the same efficiency measurements as in Fig. 4 ▸ and compares them with *CARPEM* output. Two simulation results are given: the full black line is computed with the damping factor *K* of Table 3 ▸, the dotted one with no damping (*K* = 1). The efficiency predicted by the damped model is obviously closer to the measured values. As one can observe, glitches are also better reproduced. The nonconformity from one layer to the next spreads the truncation rods of the grating in the reciprocal space thus creating larger and deeper diffraction losses – or glitches – between orders around 4250 eV. The damped curve fits very well the last point of the SIRIUS beamline measurements, which was thought spurious until then.

A second series of measurements of the high energy efficiency was made on the METROLOGIE beamline’s hard X-ray branch at SOLEIL but this time focusing on the two regions where the glitches are important: around 3250 eV and around 4250 eV. These measurements are also shown in Fig. 7 ▸. The high energy glitch is indeed found where predicted by the GRD model, and with similar amplitude. Fig. 7 ▸ also shows that the lost intensity in order 1 around 3250 eV and 4250 eV is actually leaking to another order and diffraction direction (−2 and −1, respectively).

**Figure 8:**
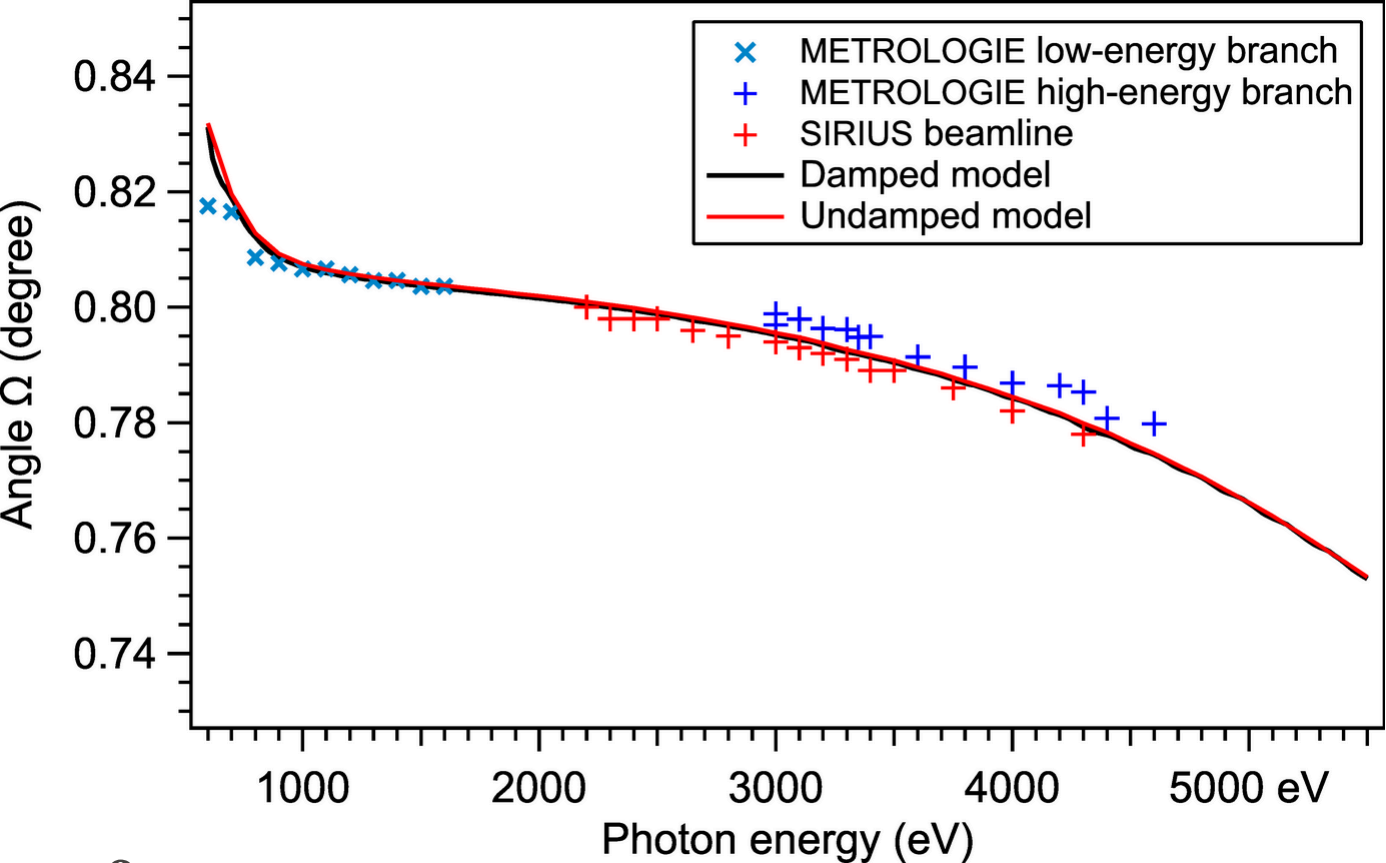
Angle Ω of peak efficiency predicted by the damped GRD model (Tables 3 ▸ and 4 ▸), the undamped model (*K* = 1) and the measured data at the METROLOGIE and SIRIUS beamlines.

The precision of the measurement of the angle Ω of peak efficiency (Fig. 8 ▸) is limited by the errors on the zero angles of the goniometer of each beamline. The two models completely agree together and are in good agreement with the measured values.

## 5. Pairing a mirror to the grating

**Figure 9:**
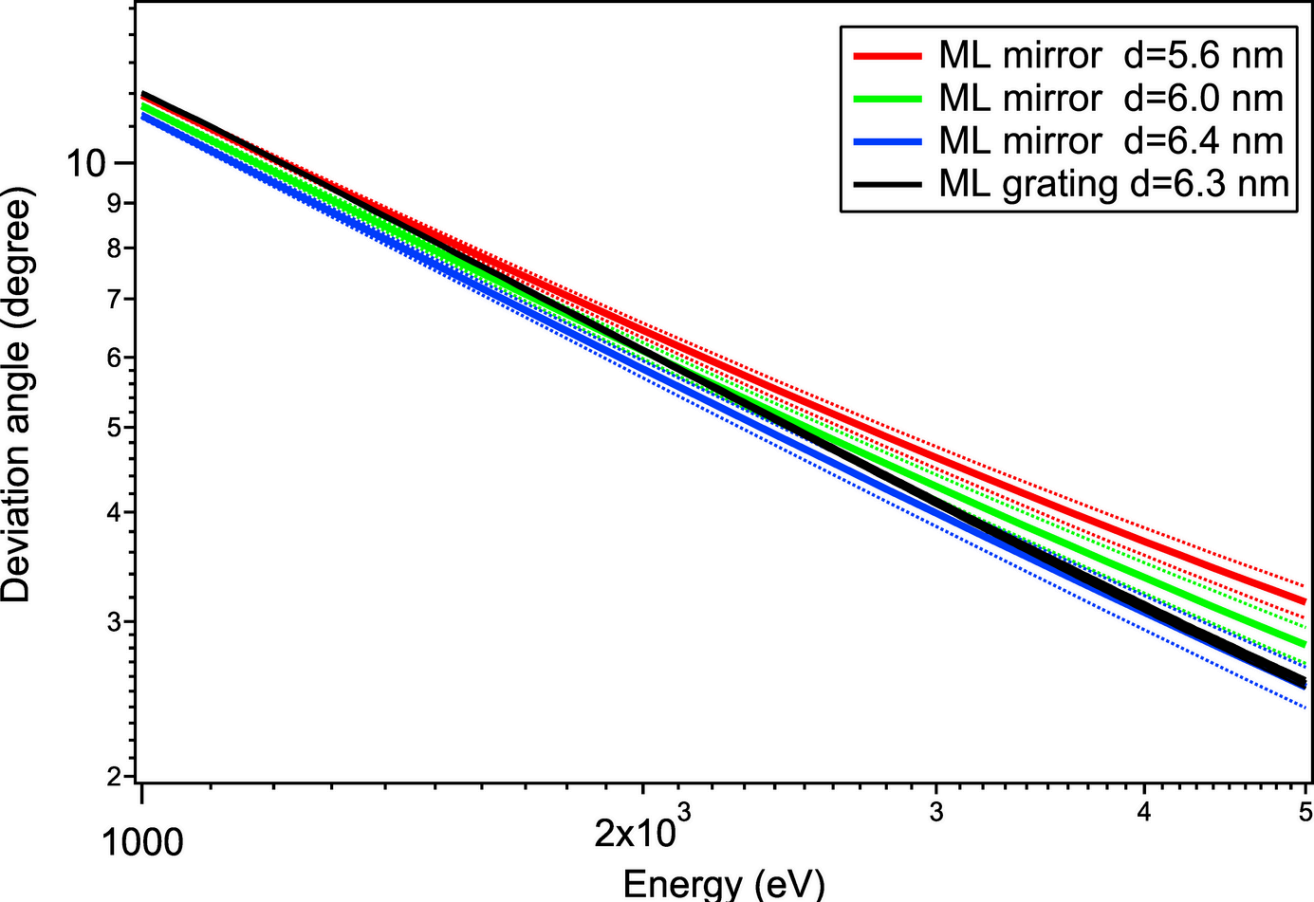
Dispersion curves of the SIRIUS grating and mirror, for three different periods of the ML mirror. Full lines locate the rocking curve maximum, while light dotted curves mark the width at half-maximum.

In synchrotron beamline monochromators, gratings are normally paired with a mirror in order to cancel out their deviation and provide a fixed exit beam. SIRIUS’s monochromator has the most usual Petersen’s configuration, where the flat grating rotates around the center of its face and the mirror holder around a precise location below the grating rotation axis (Riemer & Torge, 1983 ▸; Ciatto *et al.*, 2019 ▸). However, due to different refraction effects, a multilayer deposited on a grating does not yield the same Bragg angle as a multilayer of the same period deposited on a mirror. The period of the mirror multilayer must be adjusted, but the optimal value depends on the photon energy and the two rocking curves overlap on a limited range only, as shown in Fig. 9 ▸. Therefore, a mirror with a uniform ML period cannot cover the full range of grating efficiency.

To cope with this effect, a multilayer mirror was fabricated with a slight transverse gradient of its period. Since inside the monochromator the beam footprint is only a few millimetres wide, the optimal overlap of the two rocking curves can be achieved by the lateral translation of the mirror with a minimal efficiency loss.

**Figure 10:**
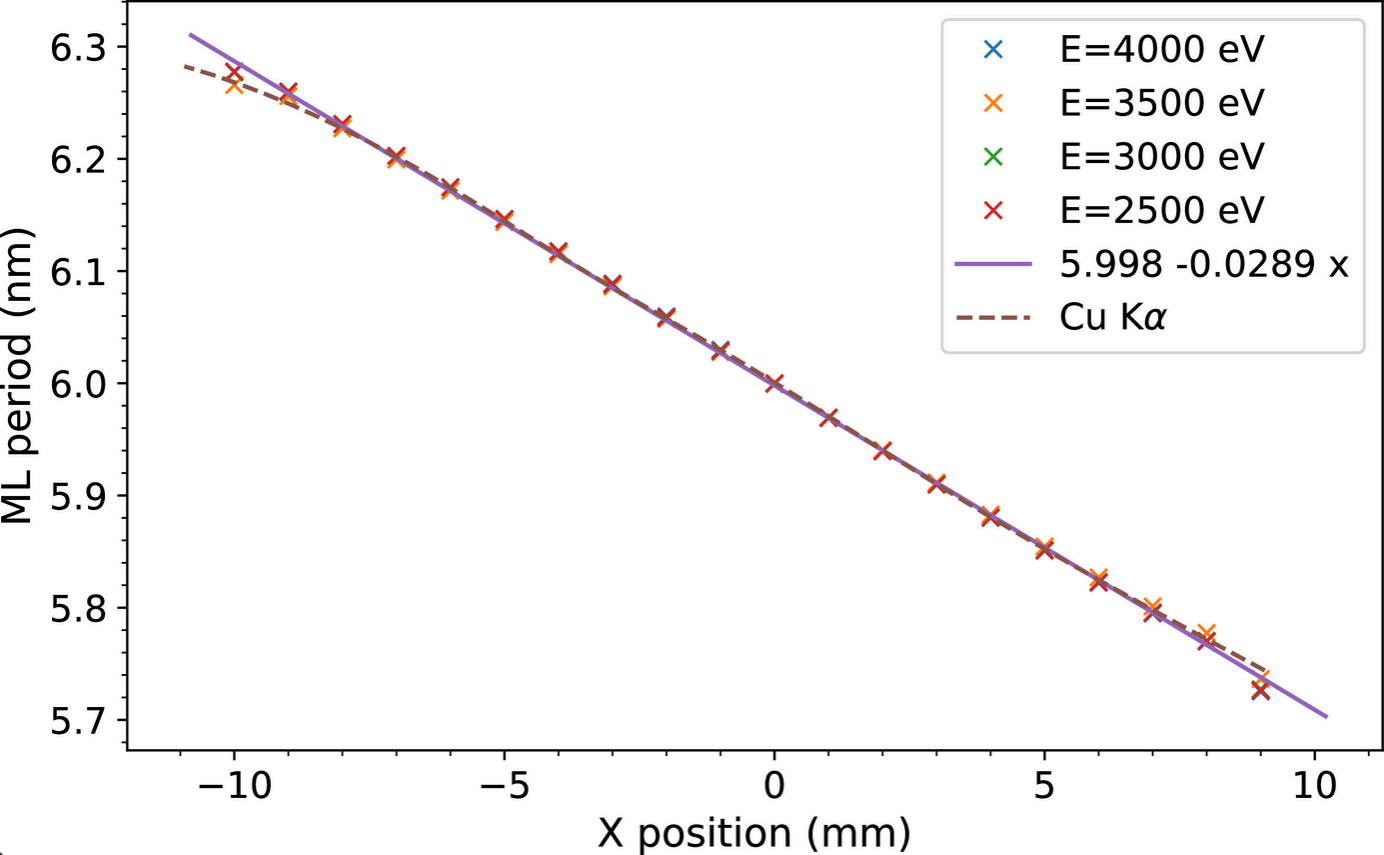
Multilayer period variation over the transverse dimension of the mirror of SIRIUS monochromator. The period is evaluated from the angular position of the peak of rocking curves measured at four different photon energies on the SIRIUS beamline, and with Cu *K*α radiation at LCF. The absolute positioning of the beam from one energy to the other is not better than ±0.3 mm so that the positions of different sets are globally adjusted.

Fig. 10 ▸ shows a period gradient of 0.0289 nm mm^−1^ (about 10% period variation on the 20 mm width) which was achieved on this mirror. The measurements of the ML period variation over the transverse dimension of the mirror were made by recording a set of reflectivity rocking curves with a position interval of 1 mm and at four energies from 2.5 to 4 keV.

## 6. Performance of SIRIUS’s monochromator

**Figure 11:**
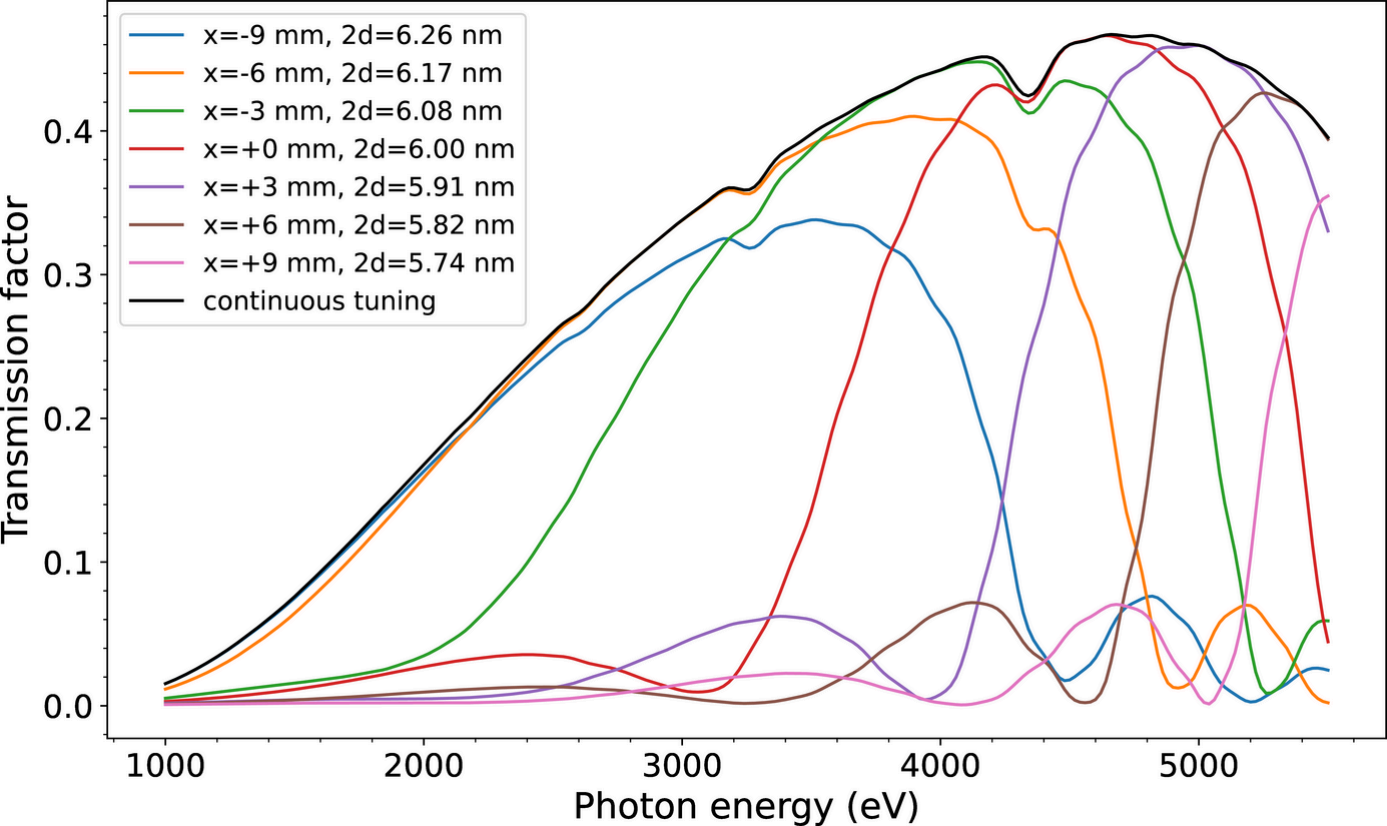
Overall transmission of the monochromator evaluated from fits of ML grating and mirror models with measured reflectivities. The transmission is plotted for seven transverse positions of the mirror spaced by 3 mm. The monochromator also allows a continuous adjustment of the mirror position with energy yielding to the black curve.

The overall transmission of the complete monochromator cannot be directly measured, and its computation from measurements only, would require a beam time consuming set of rocking curves for a large number of energies and mirror transverse positions. However, a quite satisfactory estimate of the mirror reflectivity versus energy and lateral position can be obtained by fitting the same set of rocking curves of reflectivity from which Fig. 10 ▸ is derived, with models of the ML mirror. The agreement between fitted and measured reflectivity is about 3% (at 2σ). These computations yield the transmission curves of Fig. 11 ▸. When used at a fixed position of the mirror, the bandpass is limited by the mismatch between the grating deviation and the ML mirror period. When the continuous tuning mode is used, the overall monochromator transmission factor can remain over 40% in a large energy range from 3.3 to 5.3 keV.

**Figure 12:**
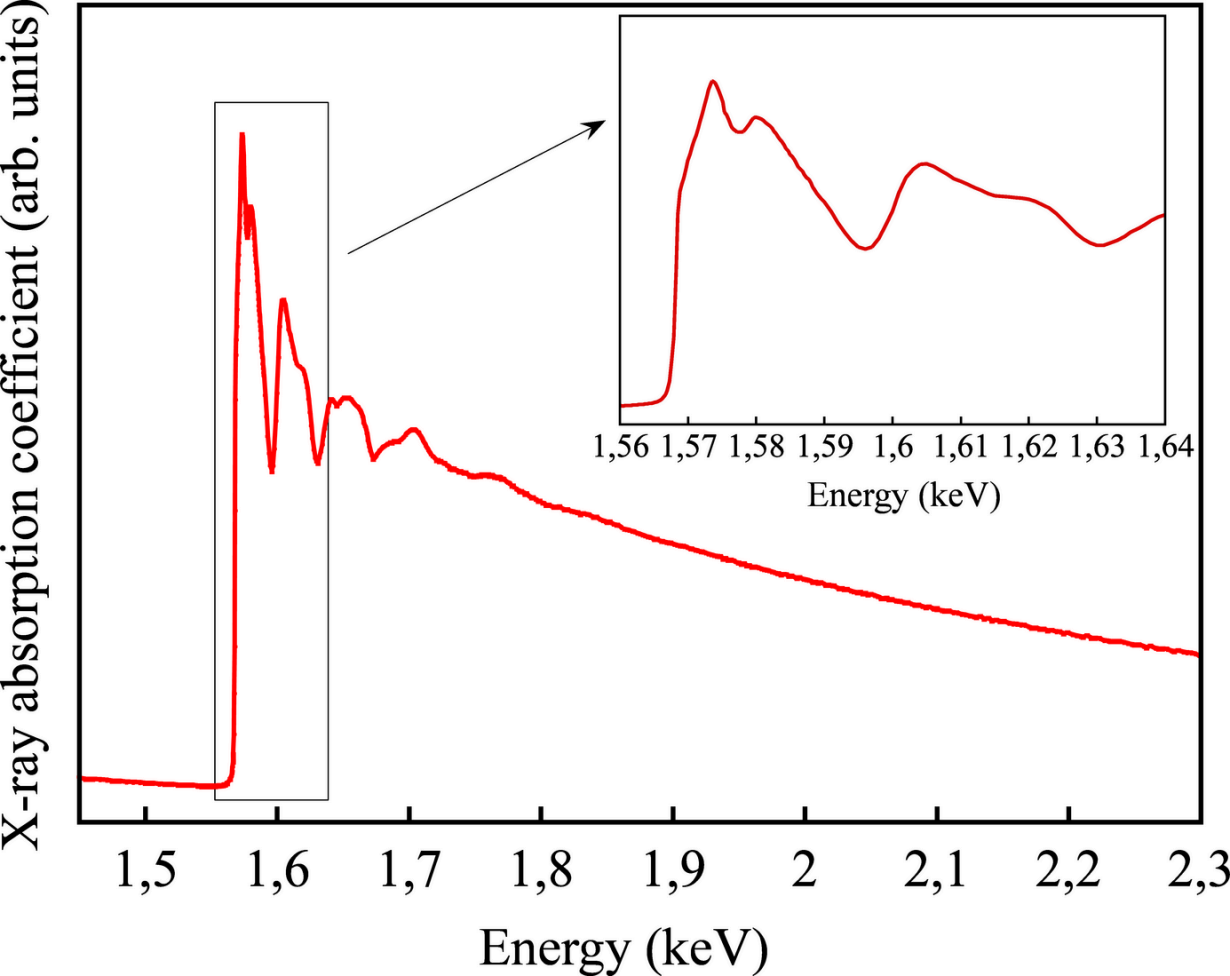
X-ray absorption spectrum of an Al foil taken in transmission mode. The main figure shows the full EXAFS spectrum, while the inset shows a zoom on the XANES region.

The grating and multilayer mirror described above have been installed in a dedicated monochromator of the SIRIUS beamline (Ciatto *et al.*, 2016 ▸) and they were commissioned in 2017. The monochromator mechanism, based on the Petersen PGM principle, allows for independent rotations of the grating and the mirror and a lateral translation of the mirror cradle. The three movements and the undulator gap are synchronized with the energy selection command enabling the acquisition of long energy scans as required in extended X-ray absorption fine structure spectroscopy (EXAFS). At the same time, the monochromator energy resolution is more than sufficient to catch the details of the X-ray absorption near-edge structure spectroscopy (XANES) region in the tender X-ray range. In Fig. 12 ▸ we show an example of an X-ray absorption spectroscopy (XAS) measurement carried out on an aluminium foil in transmission mode; the full EXAFS spectrum is shown in the main figure, while the inset zooms on the XANES region. The MLG monochromator has been used for several experiments performed at the SIRIUS beamline, for example in the study of monolayer contact doping of Si (Sgarbossa *et al.*, 2021 ▸), clustering phenomena in AlGaN semiconductors (Spindlberger *et al.*, 2023 ▸) and surface reconstructions in nanometric oxides (Etinger-Geller *et al.*, 2019 ▸).

**Figure 13:**
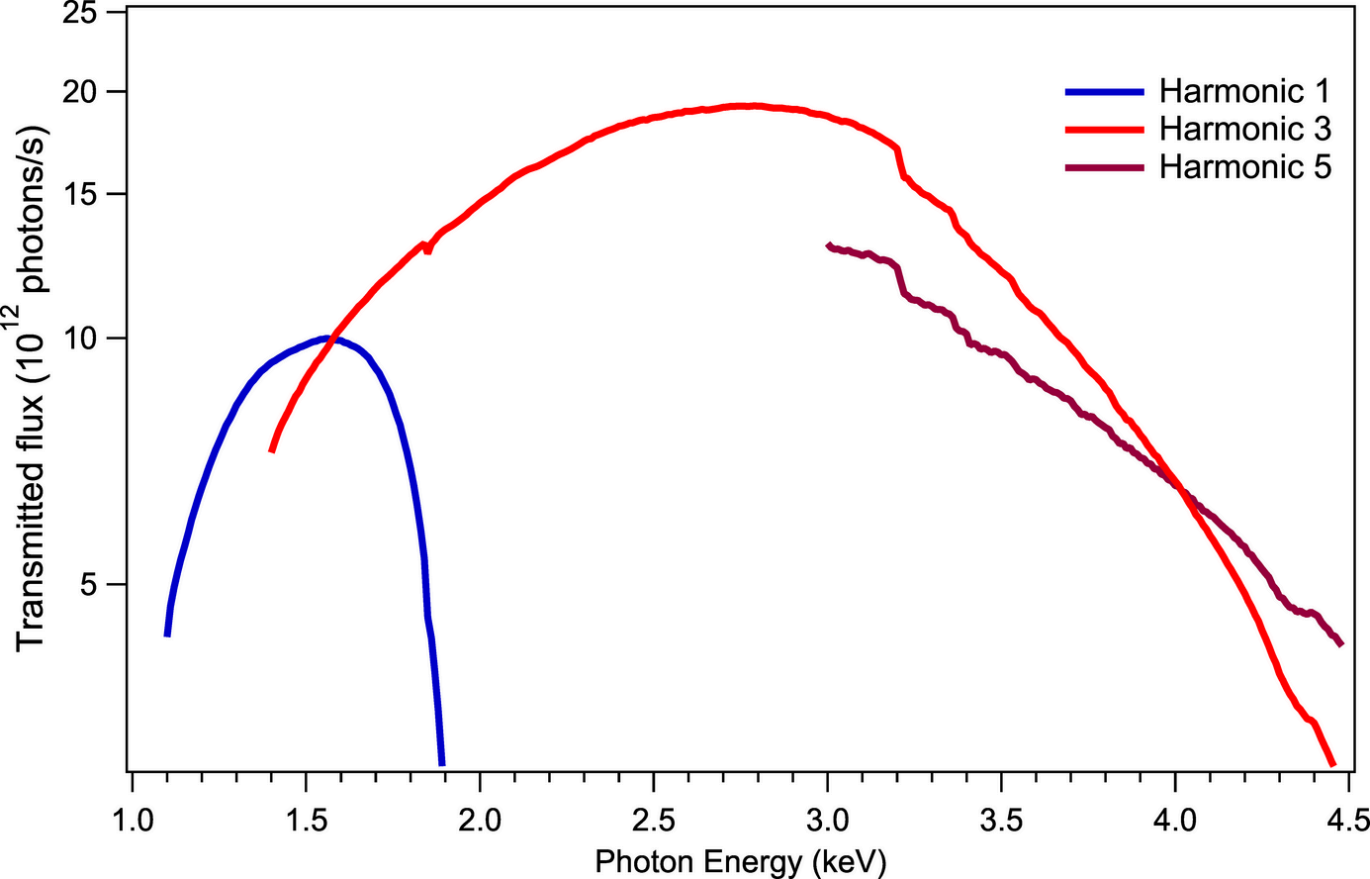
Measured SIRIUS beamline transmitted flux on sample with the MLG monochromator, in the three main harmonics of the undulator [data from Ciatto *et al.* (2019 ▸)].

The available flux at the sample position with the MGM monochromator is over 4 × 10^12^ photons s^−1^ over all the 1.2–4.5 keV energy range as shown in Fig. 13 ▸. The spectral resolution, theoretically better than 5000 with 5 µm slits, has been largely sufficient for measuring all the XANES features analyzed in our experiments to date. However, since the narrowest peaks studied up to now had a FWMH of roughly 2 eV around 2 keV, an experimental quantitative measurement of the limit resolution of the MLG monochromator based on the analysis of an XAS spectrum has not been possible.

## 7. Conclusions

We presented in this article how the concept of an ML grating has been implemented in the design of the SIRIUS beamline tender X-ray monochromator, with an ML coated plane mirror paired to the grating to compensate for its large deviation angle. A careful optimization was conducted at each step of the realization, by a feedback between design, accurate modeling and metrology. Optimization of the grating resulted from a precise determination of the groove depth and of the multilayer parameters, material density and thickness, as well as interface interdiffusion and roughness.

The optimal relationship between grating rotation angle and photon energy was determined, and the ML mirror was designed to match the resulting deviation. It is shown that perfect matching cannot be assured over the whole spectral range by a unique ML period. The ML coating of the mirror was hence designed and deposited with a slight transverse gradient, which permits to optimize the reflectivity by a lateral translation of the mirror.

The optimization was greatly improved by the ability to provide our RCWA simulation code *CARPEM* with an accurate model (GRD) of the ML structure including non-conformal deposition deduced from AFM profile measurements, and graded interfaces of the ML coating provided by Cu *K*α reflectivity retrofits with the IMD model. Great agreement with the measurements was found, both on angles and on reflectivity, when the parameters determined by these external metrologies are input into the diffraction code.

This tender X-ray monochromator has been successfully and reliably operated on the SIRIUS beamline since 2017, extending the beamline capacity from the hard down to the tender X-ray range. The concept and the optimization approach we have used can be pursued and developed with new ideas emerging in the field, such as high order blazed ML gratings or graded ML coated gratings, which may one day compete with crystals in tender and even harder X-ray monochromators.

## Figures and Tables

**Table 1:** AFM measured parameters of the Si grating substrate before coating The PARK NX20 AFM (Park Systems) was used in pure non-contact mode. The numbers in this table are statistical values from a 1.9 µm × 1.9 µm measured area. VLS groove density parameters come from the manufacturer metrology.

VLS groove density *N* = *N*_0 _(1 − *a*_1_*x*)	Groove depth	Duty cycle	Roughness
*N*_0_ = 2400 ± 1.6 mm^−1^*a*_1_ = 1.0 × 10^−4^ ± 2.6 × 10^−6^ mm^−1^	3.31 ± 0.2 nm	0.49 ± 0.03	0.049 ± 0.003 nm

**Table 2:** Parameters (thickness and density) of the three ML coating models inferred from Cu *K*α grazing incidence reflectometry and used for our *CARPEM* simulations IL stands for the interface layer of the B_4_C-on-Cr interface, *t* for the layer thickness, ρ (g cm^−3^) for the density associated with the given layer, and σ for the roughness of the interface with the layer immediately above.

Model	Substrate σ (nm)	Cr *t* (nm) / ρ / σ (nm)	IL nature	IL *t* (Å) / ρ / σ (nm)	B_4_C *t* (Å) / ρ / σ (nm)	B_2_O_3_*t* (nm) / ρ / σ (nm)
LEPTOS two-layer	0.15	2.58 / 7.19 / 0.68	—	—	3.72 / 2.0 / 0.21	0.96 / 2.8 / 0.25
LEPTOS three-layer	0.20	1.95 / 7.19 / 0.19	Cr11B8C2	0.94 / 5.92 / 0.2	3.42 / 2.0 / 0.28	0.68 / 2.8 / 0.21
IMD three-layer	0.15	1.80 / 7.19 / 0.2	Mean index	1.00 / — / 0.2	3.50 / 2.0 / 0.25	0.70 / 2.8 / 0.20

**Table 3:** Parameters of the substrate profile model and profile damping

Uncoated line height	*h*_0_ = 3.3 nm
Line duty factor	*a* = 0.45
Uncoated line damping coefficient	*k*_0_ = 0.990
Multilayer damping coefficient of equation (4) with *t* in nm	*K* = 0.9990

**Table 4:** Parameters of the multilayer specific to the GRD model (other parameters as in Table 2 ▸)

Cr thickness	2.425 nm
B_4_C thickness	3.875 nm
B_2_O_3_ thickness	0.68 nm
σ_B4C/Cr_	1.2 nm
σ of all other interfaces	0.2 nm

## Appendix A Reflectivity measurements in a plane parallel to the grating lines

When orienting the grating lines parallel to the incidence plane, the grating reciprocal vector is perpendicular to the light wavevector. The diffraction orders lie on a cone, the axis of which has the grating line direction. The configuration is therefore called conical diffraction geometry. The angular deviation of the diffracted waves is almost in the grating plane and very small. For a 2400 lines mm^−1^ grating at Cu *K*α (0.154 nm), the deviation angle of the first diffraction order is about 2 × 10^−2^ degrees. Hence, the index modulation along the wavevector is only due to the periodicity of the multilayer. The grating behaves as a relief phase grating with a surface coating whose reflectivity is that of the ML. Since all orders are accepted by the reflectometer, the measured reflectance is that of the multilayer alone.

This is not the case in the usual diffraction geometry, with the grating lines perpendicular to the incidence plane. In this case, the index modulation along the wavevector is a combination of the periodic modulations of the ML and of the grating since, at grazing incidences under a few degrees, the phase modulation along one grating period, 2π*k*_*x*_*p*, has the same order of magnitude as the phase modulation along one ML period, 2π*k*_*z*_*d*. This combination of two orthogonal modulations is responsible for the specific properties of thick multilayer gratings.

## References

[bb1] André, J., Benbalagh, R., Barchewitz, R., Ravet, M., Raynal, A., Delmotte, F., Bridou, F., Julie, G., Bosseboeuf, A., Laval, R., Soullié, G., Rémond, C. & Fialin, M. (2001). *X-ray Spectrom.* **30**, 212–215.

[bb2] Benbalagh, R., André, J., Barchewitz, R., Jonnard, P., Julié, G., Mollard, L., Rolland, G., Rémond, C., Troussel, P., Marmoret, R. & Filatova, E. O. (2005). *Nucl. Instrum. Methods Phys. Res. A* **541**, 590–597.

[bb3] Burcklen, C., Soufli, R., Dennetiere, D., Polack, F., Capitanio, B., Gullikson, E., Meltchakov, E., Thomasset, M., Jérome, A., de Rossi, S. & Delmotte, F. (2016). *J. Appl. Phys.* **119**, 125307.

[bb4] Burcklen, C., Soufli, R., Rebellato, J., Walton, C., Meltchakov, E., Rault, J. E., Gullikson, E. & Delmotte, F. (2019). *J. Nanosci. Nanotechnol.* **19**, 554–561. 10.1166/jnn.2019.16480 30327069

[bb5] Choueikani, F., Bridou, F., Lagarde, B., Meltchakov, E., Polack, F., Mercere, P. & Delmotte, F. (2013). *Appl. Phys. A* **111**, 191–198.

[bb6] Choueikani, F., Lagarde, B., Delmotte, F., Krumrey, M., Bridou, F., Thomasset, M., Meltchakov, E. & Polack, F. (2014). *Opt. Lett.* **39**, 2141. 10.1364/OL.39.002141 24686695

[bb8] Ciatto, G., Aubert, N., Lecroard, M., Engblom, C., Fontaine, P., Dubuisson, J.-M., Abiven, Y.-M., Janolin, P.-E., Kiat, J.-M., Dumont, Y., Berini, B., Fouchet, A. & Keller, N. (2019). *J. Synchrotron Rad.* **26**, 1374–1387. 10.1107/S1600577519003722 31274467

[bb7] Ciatto, G., Chu, M. H., Fontaine, P., Aubert, N., Renevier, H. & Deschanvres, J. L. (2016). *Thin Solid Films* **617**, 48–54.

[bb9] Etinger-Geller, Y., Polishchuk, I., Seknazi, E., Livne, A., Ciatto, G. & Pokroy, B. (2019). *Phys. Chem. Chem. Phys.* **21**, 14887–14891. 10.1039/c9cp00942f 31233047

[bb10] Gautier, J., Delmotte, F., Roulliay, M., Bridou, F., Ravet, M. F. & Jérome, A. (2005). *Appl. Opt.* **44**, 384–390. 10.1364/ao.44.000384 15717828

[bb11] George, M. A., Glaunsinger, W. S., Thundat, T. & Lindsay, S. M. (1990). *Thin Solid Films* **189**, 59–72.

[bb12] Hemmerle, A., Aubert, N., Moreno, T., Kékicheff, P., Heinrich, B., Spagnoli, S., Goldmann, M., Ciatto, G. & Fontaine, P. (2024). *J. Synchrotron Rad.* **31**, 162–176. 10.1107/S1600577523008810 PMC10833424 37933848

[bb13] Huang, Q., Kozhevnikov, I. V., Sokolov, A., Zhuang, Y., Li, T., Feng, J., Siewert, F., Viefhaus, J., Zhang, Z. & Wang, Z. (2020). *Opt. Express* **28**, 821–845. 10.1364/OE.28.000821 32121805

[bb14] Idir, M., Mercere, P., Moreno, T., Delmotte, A., Dasilva, P., Modi, M. H., Garrett, R., Gentle, I., Nugent, K. & Wilkins, S. (2010). *AIP Conf. Proc.* **1234**, 485–488.

[bb15] Kozhevnikov, I. V., van der Meer, R., Bastiaens, H. M. J., Boller, K. & Bijkerk, F. (2010). *Opt. Express* **18**, 16234. 10.1364/OE.18.016234 20721009

[bb16] Lagarde, B., Choueikani, F., Capitanio, B., Ohresser, P., Meltchakov, E., Delmotte, F., Krumrey, M. & Polack, F. (2013). *J. Phys. Conf. Ser.* **425**, 152012.

[bb17] Mirone, A., Delcamp, E., Idir, M., Cauchon, G., Polack, F., Dhez, P. & Bizeuil, C. (1998). *Appl. Opt.* **37**, 5816. 10.1364/ao.37.005816 18286074

[bb18] Montiel, F. & Neviere, M. (1994). *J. Opt. Soc. Am. A* **11**, 3241.

[bb19] Ohresser, P., Otero, E., Choueikani, F., Chen, K., Stanescu, S., Deschamps, F., Moreno, T., Polack, F., Lagarde, B., Daguerre, J.-P., Marteau, F., Scheurer, F., Joly, L., Kappler, J.-P., Muller, B., Bunau, O. & Sainctavit, P. (2014). *Rev. Sci. Instrum.* **85**, 013106. 10.1063/1.4861191 24517744

[bb20] Polack, F., Lagarde, B., Idir, M., Liard Cloup, A., Jourdain, E., Roulliay, M., Delmotte, F., Gautier, J. & Ravet-Krill, M.-F. (2007). *AIP Conf. Proc.* **879**, 489–492.

[bb21] Popov, E. & Nevière, M. (2001). *J. Opt. Soc. Am. A* **18**, 2886. 10.1364/josaa.18.002886 11688878

[bb22] Pradhan, P. C., Majhi, A. & Nayak, M. (2018). *J. Appl. Phys.* **123**, 095302.

[bb23] Reininger, R. (2011). *Nucl. Instrum. Methods Phys. Res. A* **649**, 139–143.

[bb24] Reininger, R. & de Castro, A. R. B. (2005). *Nucl. Instrum. Methods Phys. Res. A* **538**, 760–770.

[bb25] Riemer, F. & Torge, R. (1983). *Nucl. Instrum. Methods Phys. Res.* **208**, 313–314.

[bb26] Sgarbossa, F., Levarato, A., Carturan, S. M., Rizzi, G. A., Tubaro, C., Ciatto, G., Bondino, F., Píš, I., Napolitani, E. & De Salvador, D. (2021). *Appl. Surf. Sci.* **541**, 148532.

[bb27] Sokolov, A., Huang, Q., Senf, F., Feng, J., Lemke, S., Alimov, S., Knedel, J., Zeschke, T., Kutz, O., Seliger, T., Gwalt, G., Schäfers, F., Siewert, F., Kozhevnikov, I. V., Qi, R., Zhang, Z., Li, W. & Wang, Z. (2019). *Opt. Express* **27**, 16833–16846. 10.1364/OE.27.016833 31252903

[bb28] Spindlberger, A., Ciatto, G., Adhikari, R., Yadav, A. & Bonanni, A. (2023). *Appl. Phys. Lett.* **123**, 232101.

[bb29] Ulyanenkov, A. (2004). *Proc. SPIE* **5536**, 563302.

[bb30] Van Der Laan, G. & Thole, B. T. (1988). *Nucl. Instrum. Methods Phys. Res. A* **263**, 515–521.

[bb31] Vantelon, D., Trcera, N., Roy, D., Moreno, T., Mailly, D., Guilet, S., Metchalkov, E., Delmotte, F., Lassalle, B., Lagarde, P. & Flank, A.-M. (2016). *J. Synchrotron Rad.* **23**, 635–640. 10.1107/S1600577516000746 26917154

[bb33] Voronov, D. L., Anderson, E. H., Gullikson, E. M., Salmassi, F., Warwick, T., Yashchuk, V. V. & Padmore, H. A. (2013). *Appl. Surf. Sci.* **284**, 575–580.

[bb32] Voronov, D. L., Gawlitza, P., Cambie, R., Dhuey, S., Gullikson, E. M., Warwick, T., Braun, S., Yashchuk, V. V. & Padmore, H. A. (2012). *J. Appl. Phys.* **111**, 093521.

[bb34] Voronov, D. L., Gullikson, E. M., Salmassi, F., Warwick, T. & Padmore, H. A. (2014). *Opt. Lett.* **39**, 3157. 10.1364/OL.39.003157 24876001

[bb35] Voronov, D. L., Salmassi, F., Meyer-Ilse, J., Gullikson, E. M., Warwick, T. & Padmore, H. A. (2016). *Opt. Express* **24**, 11334. 10.1364/OE.24.011334 27410064

[bb36] Wen, S., Huang, Q., Sokolov, A., Zhuang, Y., Lemke, S., Seliger, T., Yu, Y., Viefhaus, J., Qi, R., Zhang, Z. & Wang, Z. (2024). *Opt. Laser Technol.* **168**, 109979.

[bb37] Wen, S., Kozhevnikov, I. V., Huang, Q., Huang, S., Zhang, Z., Hu, H., Zhang, Z. & Wang, Z. (2025). *Opt. Express* **33**, 5989. 10.1364/OE.550725 40797878

[bb38] Werner, S., Guttmann, P., Siewert, F., Sokolov, A., Mast, M., Huang, Q., Feng, Y., Li, T., Senf, F., Follath, R., Liao, Z., Kutukova, K., Zhang, J., Feng, X., Wang, Z., Zschech, E. & Schneider, G. (2023). *Small Methods* **7**, 2201382. 10.1002/smtd.202201382 36446642

[bb39] Windt, D. L. (1998). *Comput. Phys.* **12**, 360–370.

[bb40] Wu, J.-L., Qi, R., Huang, Q., Feng, Y., Wang, Z. & Xin, Z. (2019). *Chin. Phys. Lett.* **36**, 120701.

[bb42] Yang, X., Kozhevnikov, I. V., Huang, Q., Wang, H., Hand, M., Sawhney, K. & Wang, Z. (2017*a*). *Opt. Express* **25**, 15987. 10.1364/OE.25.015987 28789109

[bb41] Yang, X., Kozhevnikov, I. V., Huang, Q. & Wang, Z. (2015). *J. Opt. Soc. Am. B* **32**, 506.

[bb43] Yang, X., Wang, H., Hand, M., Sawhney, K., Kaulich, B., Kozhevnikov, I. V., Huang, Q. & Wang, Z. (2017*b*). *J. Synchrotron Rad.* **24**, 168–174. 10.1107/S1600577516017884 PMC5182023 28009556

